# Synthesis of Large-Area WS_2_ monolayers with Exceptional Photoluminescence

**DOI:** 10.1038/srep19159

**Published:** 2016-01-13

**Authors:** Kathleen M. McCreary, Aubrey T. Hanbicki, Glenn G. Jernigan, James C. Culbertson, Berend T. Jonker

**Affiliations:** 1 Naval Research Laboratory, Washington DC 20375, USA

## Abstract

Monolayer WS_2_ offers great promise for use in optical devices due to its direct bandgap and high photoluminescence intensity. While fundamental investigations can be performed on exfoliated material, large-area and high quality materials are essential for implementation of technological applications. In this work, we synthesize monolayer WS_2_ under various controlled conditions and characterize the films using photoluminescence, Raman and x-ray photoelectron spectroscopies. We demonstrate that the introduction of hydrogen to the argon carrier gas dramatically improves the optical quality and increases the growth area of WS_2_, resulting in films exhibiting mm^2^ coverage. The addition of hydrogen more effectively reduces the WO_3_ precursor and protects against oxidative etching of the synthesized monolayers. The stoichiometric WS_2_ monolayers synthesized using Ar + H_2_ carrier gas exhibit superior optical characteristics, with photoluminescence emission full width half maximum (FWHM) values below 40 meV and emission intensities nearly an order of magnitude higher than films synthesized in a pure Ar environment.

Single- to few-monolayer transition metal dichalcogenides (TMDs) hold promise for technological applications in a variety of areas including photodetection^1^, flexible electronics^2^,^3^, and chemical sensing^4^. While MoS_2_ has received the most attention due to its relative ease of mechanical exfoliation^5^,^6^,^7^,^8^,^9^,^10^,^11^, the closely related WS_2_^12^,^13^,^14^, MoSe_2_^15^,^16^, and WSe_2_^17^,^18^ are quickly gaining notice. Recently, WS_2_ demonstrated superior optical properties compared to MoS_2_ as measured by luminescent quantum efficiency and linewidths^12^,^19^,^20^. In addition, WS_2_ has larger spin-orbit coupling^21^, suggesting WS_2_ may exhibit larger band edge spin splittings and stronger magnetic field effects for optoelectronic and spintronic functionalities.

Weak inter-layer van der Waals bonding enables single layers of TMD materials to be easily isolated using mechanical exfoliation in order to probe fundamental optical, electronic, and spintronic properties^22^. Unfortunately, deposited flakes tend to be randomly positioned and irregularly shaped, posing significant challenges for integration into technological applications. A cost effective and reliable means to achieve large-area, high quality materials is a foundational step for the incorporation of monolayer WS_2_ into future technologies.

Chemical vapor deposition (CVD) has proven successful in the synthesis of wafer scale monolayer graphene material^23^,^24^ and is showing promise in WS_2_ synthesis^19^,^20^,^25^,^26^,^27^,^28^,^29^,^30^,^31^,^32^,^33^. CVD synthesis of monolayer TMD materials can be performed in a quartz tube furnace under the flow of an inert gas. In the case of WS_2_, WO_3_ and S precursors are used. The sulfur vapor partially reduces the WO_3_ at elevated temperatures to form a volatile WO_3-x_ species, which adsorbs onto the growth substrate and subsequently reacts with sulfur to produce WS_2_. While several independent research groups have reported successful WS_2_ synthesis using WO_3_ and S precursors, specific details of the procedure (precursor amount, growth substrate, growth pressure, temperature, gases, flow rates, etc.) can vary widely from lab to lab. Subsequently, properties such as growth morphology, luminescence yield, and Raman spectra exhibit considerable variations.

To better understand and improve the quality of CVD synthesized monolayer WS_2_, it is important to investigate materials grown under various conditions in a single, well-controlled CVD system. In this work, we demonstrate that small modifications to temperature and carrier gas have substantial effects on the resulting coverage, continuity, and quality of CVD synthesized WS_2_ monolayers. By optimizing the growth conditions, we achieve large-area (~mm^2^) monolayer WS_2_ with superior optical characteristics. Additionally, by introducing hydrogen gas during synthesis, we are able to prevent the oxidation of WS_2_, which may also be relevant to synthesis of other TMD monolayers and van der Waals heterostructures^34^.

## Results and Discussion

**Figure 1:**
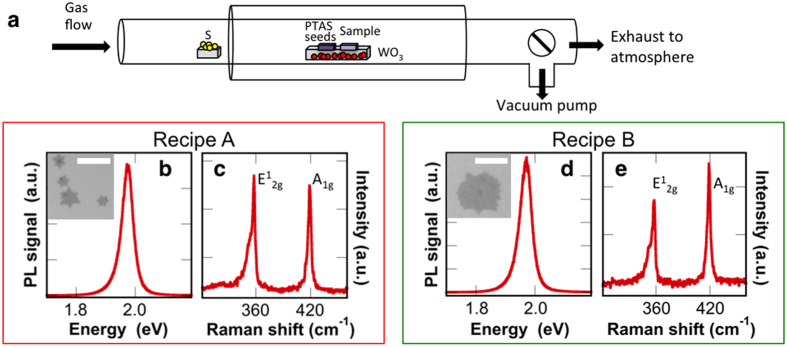
**(a) Schematic of the quartz tube furnace used for WS_2_ monolayer synthesis.** (b) Photoluminescence and (c) Raman spectra confirm the identity and monolayer nature of WS_2_ synthesized under recipe (A). An optical microscope image (inset of b) displays isolated, monolayer growth with star-like configuration. (d) Photoluminescence and (e) Raman spectra of monolayer WS_2_ synthesized with recipe (B). The inset of (d) displays an optical image of the irregular, isolated WS_2_ monolayer growth. Scale bars are 10 μm.

Synthesis of monolayer WS_2_ is performed in a quartz tube furnace, as shown in Fig. 1a. At the center of the furnace is positioned a quartz boat containing ~1 g of WO_3_ powder. Two Si/SiO_2_ (275 nm) wafers are positioned face-down, directly above the precursor. The upstream wafer contains perylene-3,4,9,10-tetracarboxylic acid tetrapotassium salt (PTAS) seeding molecules, while the downstream substrate is untreated. The hexagonal PTAS molecules are carried downstream to the untreated substrate and promote lateral growth of the TMD materials^35^. While WS_2_ growth occurs on both substrates, the downstream (untreated) substrate is the focus of this manuscript. Additional details regarding PTAS preparation and use can be found in the supplementary information. Sulfur is placed upstream, outside the furnace heating zone. It should be noted that great care is taken to position the WO_3_ precursor, substrates, and the sulfur source at identical positions for each growth, as the positioning may affect the growth dynamics and resulting film quality^27^. Prior to synthesis, a pump-flush procedure is performed in the quartz tube to promote a uniform initial environment that aids in the reproducibility. The quartz tube is evacuated to 100 mTorr then refilled with Ar gas until atmospheric pressure is reached. This cycle is repeated twice before a continuous flow of Ar is utilized for growth. All recipes are performed at atmospheric pressure.

The initial growth (recipe A) is performed under continuous 100 sccm argon flow. The furnace temperature is quickly ramped to 625 **°**C at a rate of 20 **°**C/min followed by a 10 **°**C/min ramp to 825 **°**C. At this point, the temperature is held constant for 10 minutes and then allowed to cool. This procedure produces isolated islands exhibiting lateral dimensions of several μm, often with star-like orientations (inset of Fig. 1b). Small areas of thicker growth are observed on some WS_2_ islands. Similar features have been previously observed in synthesized TMD materials^11^,^36^. This characteristic growth of isolated WS_2_ islands occurs across several mm of the growth substrate and has been reproduced in multiple growth runs.

Photoluminescence (PL) and Raman spectroscopy confirm the identity and monolayer nature of the synthesized crystals. Raman spectroscopy and PL measurements are performed at room temperature in air using 488 nm laser excitation with intensity below 200 μW for a <1μm diameter spot size, to prevent sample damage. Photoluminescence measurements (Fig. 1b) reveal a sharp emission peak with maximum intensity achieved at 1.97 eV in the investigated range from 1.55 eV to 2.50 eV. The peak energy and observation of a single, intense emission peak indicates monolayer WS_2_ material. Additional layers (not shown) dramatically decrease the emission intensity and are accompanied by a low energy peak, caused by the transition to indirect bandgap semiconductor. The Raman spectroscopy measurements (Fig. 1c) detect the in-plane and out-of plane phonon modes, E^1^_2g_ and A_1g_, at 357.5 cm^−1^ and 419 cm^−1^, respectively. Additionally, the longitudinal acoustic mode at the M point, LA(M), is evident at ~350 cm^−1^, forming a low wavenumber shoulder on the E^1^_2g_ peak. The peak separation (Δk) between E^1^_2g_ and A_1g_ is commonly used to identify the layer number of WS_2_, with reported Δk values of ~62 cm^−1^ for monolayer and ~64 cm^−1^ for bilayer^36^. We measure a peak separation of 61.5 cm^−1^, confirming monolayer WS_2_ synthesis.

It is instructive to investigate the effect of temperature, as a wide range of values have been reported for WS_2_ synthesis. Upon increasing the growth temperature to 875 **°**C (recipe B), the resulting WS_2_ islands exhibit a moderate increase in lateral size, and the shape becomes more irregular (inset of Fig. 1d). Equilateral triangular growth is suggestive of single crystallinity, while star-like structures (as seen in inset of Fig. 1b) are produced from several rotationally symmetric grains^37^. Hence, we speculate that the observed change in Fig. 1d may indicate additional grain boundaries or increased defect density. Regardless of the differing growth features, the PL again displays a single sharp emission peak centered at 1.97 eV (Fig. 1d). Furthermore, the dominant E^1^_2g_ and A_1g_ Raman peaks remain at 357.5 cm^−1^ and 419 cm^−1^ (Fig. 1e), resulting in a Δk of 61.5 cm^−1^ and indicative of single monolayer growth. Growth at higher temperatures (975 **°**C in Ar flow) dramatically reduces WS_2_ growth, resulting in very little substrate coverage. This observation suggests that conditions at elevated temperatures may be detrimental to WS_2_, and is discussed further in the supplementary material.

**Figure 2:**
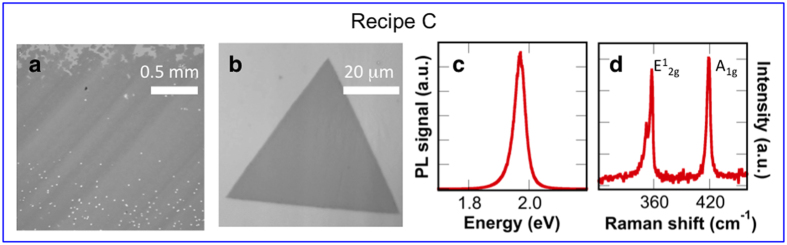
Characterization of WS_2_ synthesized using recipe C: (a) The optical image displays large areas (~1 mm × 1 mm) of continuous monolayer WS_2_ (dark grey). The edge of the film is visible at the top of the image, where areas of bare substrate are evident (light grey). Few sites of multilayer growth are evident in the bottom half of the image (white specs). (b) An image of an isolated, equilateral WS_2_ monolayer. (c) Photoluminescence and (d) Raman spectra, confirming monolayer WS_2_.

The effects of carrier gas are summarized in Fig. 2. Once the growth temperature of 825 **°**C is reached, 10 sccm hydrogen is added to the 100 sccm Ar flow, and continues throughout the completion of the recipe (recipe C). The introduction of hydrogen produces substantially different growth characteristics compared to the isolated islands synthesized in recipes A and B. WS_2_ islands now coalesce to form nearly continuous films on the mm scale (Fig. 2a). Optical and AFM images confirm the film is composed primarily of monolayer WS_2_, with multilayer growth constituting less than 8% of the growth area. Additional details are provided in the supplementary materials. Continuous monolayer growth on the mm scale is typical of samples synthesized using recipe C. Figure 2b shows an individual WS_2_ crystal, at the edge of the coalesced film. Triangular growth is observed with lateral dimension larger than 50 μm, nearly an order of magnitude larger than isolated WS_2_ islands synthesized in pure argon at an equivalent temperature. Even though recipe C results in dramatically different growth morphology, PL and Raman spectra exhibit characteristics similar to previous recipes (Fig. 1b–d), with a single sharp PL emission at ~1.97 eV (Fig. 2c) and E^1^_2g_ and A_1g_ Raman peaks measured at 357.5 cm^−1^ and 419 cm^−1^ (Fig. 2d).

**Figure 3:**
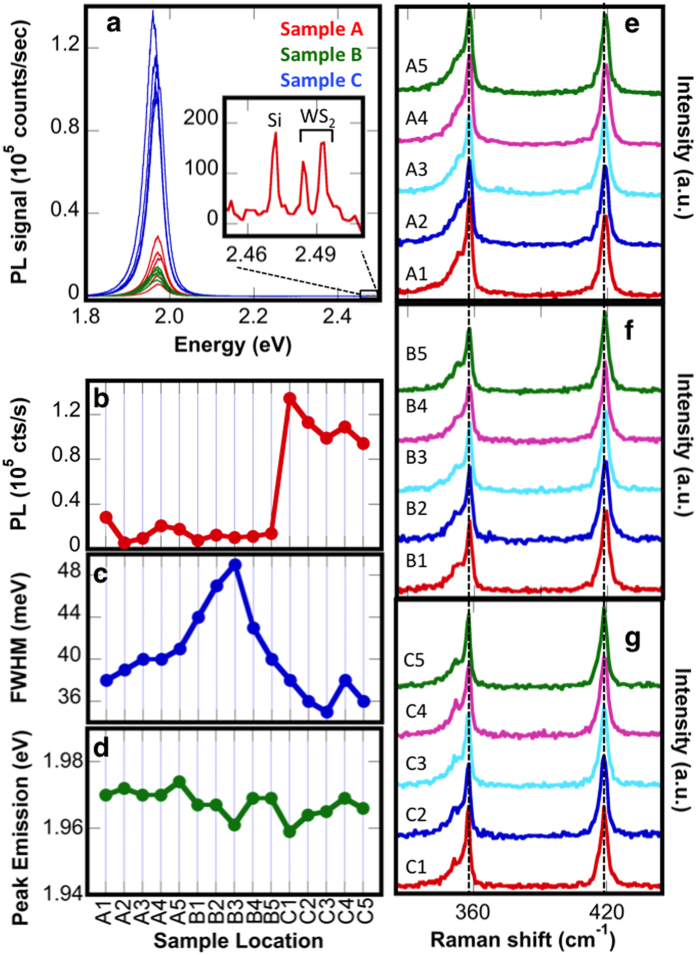
Comparisons of monolayer WS_2_ synthesized under different recipes. (a) WS_2_ synthesized using recipe C repeatedly results in the highest intensity PL emission. The Raman peaks from WS_2_ and the Si substrate are also measureable (inset). The large PL/Raman ratio demonstrates the high luminescent efficiency. Values for (b) PL intensity, (c) full width at half maximum, and (d) PL peak position are shown for multiple randomly selected monolayer locations on a given sample. (e–g) The corresponding Raman spectra are displayed. The E^1^_2g_ and A_1g_ Raman peaks are observed at 357.5 cm^−1^ and 419 cm^−1^ for all WS_2_, as indicated by dashed vertical lines.

The monolayer films synthesized using procedures A, B and C just described are further investigated by obtaining multiple Raman and PL measurements across large areas of the growth wafer. On each wafer, 5 measurement sites have been randomly selected, with each location more than 500 μm away from the closest neighbor. Additionally, only monolayer regions substantially larger than the laser spot size and free of visible multilayer growth were selected. Therefore, any measured differences are attributed to WS_2_ quality as opposed to sample size, edge effects, or overlayer growth. While photoluminescence measurements resulted in qualitatively similar spectra, it is immediately apparent in Fig. 3a, the emission intensity is extremely sensitive to the growth recipe. Samples synthesized using recipe C consistently result in the highest PL intensity, with values ranging from 94,300 to 134,300 counts/second. Recipes A and B have considerably lower intensities, with sample A ranging from 5,700 to to 28,400 counts/sec and B from 7,800 to 14,000 counts/sec (Fig. 3b).

In addition to the dominant PL emission peak, two Raman peaks (E^1^_2g_, A_1g_) plus that of the Si substrate are evident at higher energy (inset of Fig. 3a). The relation between PL and Raman intensity has been utilized as a metric to gauge optical quality of TMD materials and determine intrinsic luminescence quantum efficiency^6^. Higher PL/Raman ratios signify higher optical quality. Measurements for monolayer MoS_2_ report a moderate PL/Raman ratio of 10 or less^6^,^37^,^38^,^39^. Recent studies of monolayer WS_2_ report a wide range of luminescence quantum efficiencies, ranging from 1–10 to several hundred^19^,^20^,^40^. Our WS_2_ monolayers synthesized with recipes A and B demonstrate PL/Raman ratios ranging from 20 to 120, whereas WS_2_ synthesized under recipe C demonstrates ratios above 450 and as high as 650.

A further gauge of sample quality is the PL FWHM, with smaller FWHM indicative of higher optical quality. While all recipes exhibit FWHM below 50 meV, as previously reported for high quality WS_2_ monolayers, it is clear that recipe C demonstrates the lowest FWHM (Fig. 3c), with values as low as 35 meV corresponding to the high PL intensities measured. Conversely, samples synthesized using recipe B exhibit the highest FWHM values, consistent with the low crystalline order suggested by optical and AFM images. Although substantial variations are observed in PL intensity and FWHM, the PL peak position exhibits only minor deviations from 1.97 eV, as shown in Fig. 3d.

The corresponding Raman spectra measured at each site are plotted for the various samples (Fig. 3e–g). Independent of recipe, the E^1^_2g_ and A_1g_ peaks remain constant at 357.5 cm^−1^ and 419 cm^−1^ for all WS_2_, as indicated by the dashed line provided as a guide to the eye. While the peak positions exhibit little variation, we do observe small modifications in the relative intensities between E^1^_2g_ and A_1g_. Samples synthesized under recipe A tend to have a slightly higher E^1^_2g_ intensity, with the ratio E^1^_2g_/A_1g_ ranging from 1.0 to 1.2. Conversely the A_1g_ intensity is higher for recipe B, with E^1^_2g_/A_1g_ from 0.7 to 0.9. The samples synthesized with recipe C exhibit nearly equal intensities with E^1^_2g_/A_1g_ ~0.9 to 1.0. While moderate changes in the Raman intensity ratio are known to indicate a change in layer number for exfoliated WS_2_^36^,^41^, we exclude this as the source of variation in our synthesized materials, as no increase in peak separation is observed, and independent techniques (optical microscopy, atomic force microscopy, PL) show no sizeable multilayer growth in the surveyed areas. Alternatively, minor intensity variations in E^1^_2g_ and A_1g_ peaks of monolayer TMDs indicate differences in electronic doping levels or strain^42^,^43^. We hypothesize that the various recipes result in differences in the sulfur content of the films, which may affect both doping and local strain and contribute to the observed intensity variations, although additional investigations are necessary to confirm these speculations.

**Figure 4:**
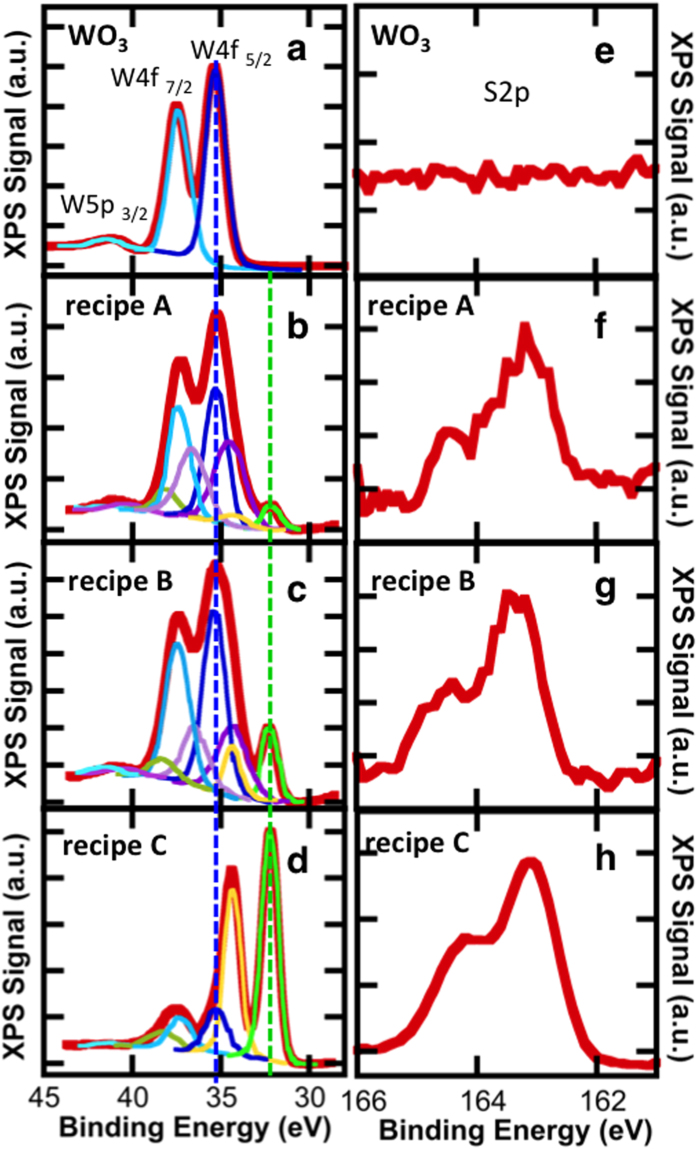
Chemical analysis using XPS. (a–d) Spectra of the tungsten core levels. (a) The WO_3_ precursor powder (red line) is fit by three peaks (blue) corresponding to W4f_7/2_, W4f_5/2_, and W5p_3/2_ core levels. The binding energies indicate a (6+) valence state, as expected for oxidized tungsten. (b,c) Spectra from recipes A and B exhibit WO_3_ peaks as well as two additional sets of peaks. The additional peaks signify the presence of partially reduced WO_3_ (purple lines), and oxygen-free tungsten in the (4+) valence state (green/yellow lines). (d) For recipe C, oxygen-free tungsten peaks dominate. Partially reduced WO_3_ is no longer present and WO_3_ is considerably reduced. As a guide to the eye, dashed lines indicate the W4f _5/2_ binding energy in the 6+ (blue) and 4+ valence state (green). (e–h) XPS spectra of sulfur core levels. (e) No sulfur is present in the WO_3_ precursor. (f–h) The S2p doublet confirms the presence of WS_2_ for samples synthesized using recipes A–C.

To further understand the relative differences in sample characteristics resulting from these growth processes, X-ray photoelectron spectroscopy (XPS) is used to determine the chemical composition and stoichiometry of our WS_2_ films. Spectra are obtained with monochromated Al Kα radiation using a 400 μm aperture. Figure 4 shows the XPS spectra of the tungsten and sulfur core levels in the WO_3_ precursor as well as WS_2_ monolayers synthesized using recipes A, B, and C. The WO_3_ precursor powder (Fig. 4a, red line) is well fit by three peaks at binding energies 35.3 eV, 37.4 eV, and 41.3 eV corresponding to W4f_7/2_, W4f_5/2_, and W5p_3/2_ core energy levels, respectively. The binding energies indicate the valence state of the material and are consistent with a (6+) valence state^44^,^45^, as expected for oxidized tungsten. A decrease in valence due to S- rather than O-bonding will be indicated by a shift in the W4f and W5p core levels to lower energies. The (6+) valence remains the dominant contribution in samples fabricated using both recipe A (Fig. 4b) and recipe B (Fig. 4c), indicating a large amount of WO_3_ in these synthesized monolayers. In both samples, two additional sets of peaks are present, demonstrating the presence of partially reduced WO_3_ (purple lines), as well as oxygen-free tungsten in the (4+) valence state (green/yellow lines), as expected for WS_2_. The sulfur S2p peak in samples A and B (Fig. 4f,g) consists of a single doublet corresponding to S-W bonding, and confirms the presence of WS_2_. The considerably high oxygen content in these samples is surprising however, particularly in light of the photoluminescence and Raman characteristics that are comparable to high quality WS_2_. This highlights the importance of sensitive chemical analysis techniques, such as XPS, in the rapidly progressing field of 2D materials synthesis.

The addition of hydrogen in recipe C results in a noticeable change in the chemical composition. In the tungsten core levels, the (4+) valence state becomes the dominant contribution (Fig. 4d). Peaks indicative of partially reduced WO_3_ are no longer present, and the WO_3_ (6+valence) is considerably reduced. A strong S2p doublet is observed, as evident in Fig. 4h. The improved sulfur signal is primarily due to the increased growth area present in these samples. Analysis of the S2p doublet and W4f peaks associated with WS_2_ (the (4+) valence state) provide a S:W ratio of 2, indicating stoichiometric WS_2_ synthesis for samples produced using recipe C. On the contrary, samples synthesized using recipes A and B exhibit a large sulfur deficiency, having only 45% sulfur content. Instrumentation limitations and fitting procedures give rise to a conservative error of less than 5% in the W/S ratio. It is clear that the addition of hydrogen during synthesis leads to more efficient reduction of the WO_3_ precursor and conversion to WS_2_. This has the multiple benefit of resulting in larger synthesis areas, improved chemical composition, and significant improvements in the optical properties. Furthermore, performing post-growth anneals in pure Ar or Ar/H_2_ environments show that hydrogen aids in suppressing oxidative etching that stems from unintended leaks in the CVD system. These findings are discussed in detail in the supplementary information.

## Conclusion

In conclusion, we have demonstrated synthesis of monolayer WS_2_ under various conditions. Optical characterization using photoluminescence and Raman spectroscopy of the various WS_2_ samples show that the addition of hydrogen into the flow stream produces significantly higher PL intensity and narrower linewidths. XPS studies demonstrate that this is due to the formation of high-quality, low oxygen content monolayers achieved only when hydrogen is introduced during synthesis. The addition of hydrogen more effectively reduces the WO_3_ precursor, resulting in monolayer films exhibiting mm^2^ lateral dimensions, while simultaneously protecting against oxygen damage.

## Additional Information

**How to cite this article**: McCreary, K. M. *et al.* Synthesis of Large-Area WS_2_ monolayers with Exceptional Photoluminescence. *Sci. Rep.* **6**, 19159; doi: 10.1038/srep19159 (2016).

## Supplementary Material

Supplementary Information
