# Supplementary material for: Synthesis of Large-Area WS2 monolayers with Exceptional Photoluminescence
Source: Sci Rep. 2016 Jan 13;6:19159. doi: 10.1038/srep19159 (PMC4725944; doi:10.1038/srep19159)
Supplement: Supplementary Information [file srep19159-s1.pdf]

Supplementary Information

Title: **Synthesis of Large-Area WS_2 monolayers with Exceptional Photoluminescence**

Kathleen M. McCreary, Aubrey T. Hanbicki, Glenn G. Jernigan, James C. Culbertson, Berend T. Jonker*

We utilize Perylene-3,4,9,10-tetracarboxylic acid tetrapotassium salt (PTAS) to promote the growth of WS_2 on Si/SiO_2 (275 nm) substrates.[1] A ~1mM liquid solution is established by dissolving 17 mg PTAS in 30 mL water. (PTAS molecular weight = 580.7 g/mol). Approximately 30 mL of the PTAS solution is pipetted onto a clean Si/SiO_2 substrate, wetting the surface. The substrate is subsequently heated to 85 °C on a hotplate in ambient conditions until the water is fully evaporated. The PTAS sample is then loaded into the quartz tube face-down, directly above the WO_3 precursor and upstream of the bare growth substrate. During CVD synthesis, PTAS seeds are carried downstream to stimulate the growth of monolayer WS_2. XPS confirms the presence of a small amount of PTAS on the downstream growth substrate following all synthesis recipes (Recipe A, B, and C discussed in the main text). Small variations in concentration are measured, with samples synthesized using recipe A exhibiting the largest concentration of PTAS and recipe C the smallest.

Synthesis of WS_2 in the absence of PTAS seeds is also investigated. In this case, two bare Si/SiO_2 (275 nm) substrates are loaded face-down, directly above the WO_3 precursor. Synthesis conditions identical to recipes A and B result in no growth on either substrate. Synthesis conditions identical to recipe C result in WS_2 growth on both the upstream (Figure S1) and downstream (Figure S2) substrates. Although, in the absence of PTAS seeds, the morphology and uniformity are considerably different than when PTAS is utilized. Only isolated island growth,

having lateral dimensions up to tens of μm, is observed. The synthesized islands are commonly a rounded-triangle shape, with a large amount of multilayer WS_2 in addition to monolayer growth. While monolayer synthesis is possible in the absence of PTAS (under certain conditions), the growth area and uniformity are significantly improved when PTAS is utilized.

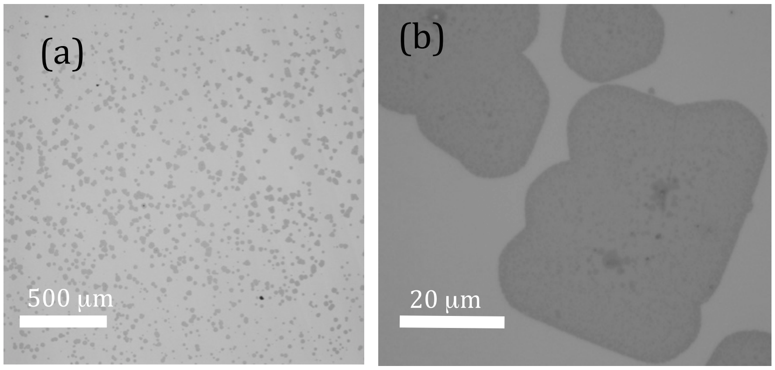

Figure S1: Optical images of WS_2 synthesized on the **upstream** substrate *without* the aid of PTAS seeds. (a) For synthesis conditions identical to recipe C, only isolated island growth occurs. (b) The high-magnification image shows monolayer growth in rounded-triangle shapes with a considerable amount of multilayer growth (darker grey contrast).

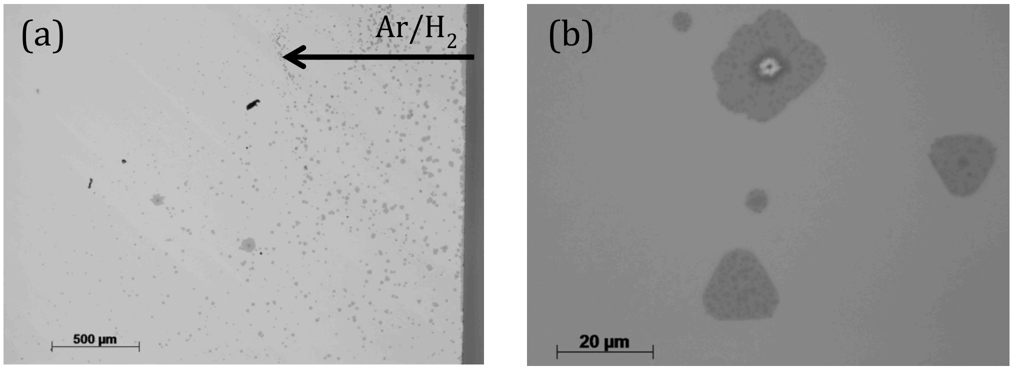

Figure S2: Optical images of WS_2 synthesized on the **downstream** substrate *without* the aid of PTAS seeds. (a) For synthesis conditions identical to recipe C, isolated island growth is observed within a few mm of the upstream edge. The flow direction is indicated for clarity. (b) Rounded shapes are evident in the high-magnification image, as well as multilayer WS_2 growth (darker grey contrast).

Atomic force microscopy is utilized to probe the uniformity and structure of WS_2 synthesized under the various recipes. As discussed in the main text, both recipes A (pure Ar,

T=825 °C) and recipe B (pure Ar, T=875°C) result in isolated islands growth with lateral dimensions several tens of microns or less. The AFM images confirm smooth monolayer growth composes the majority of the growth region for both recipes, with only a small amount of bilayer or multilayer growth.

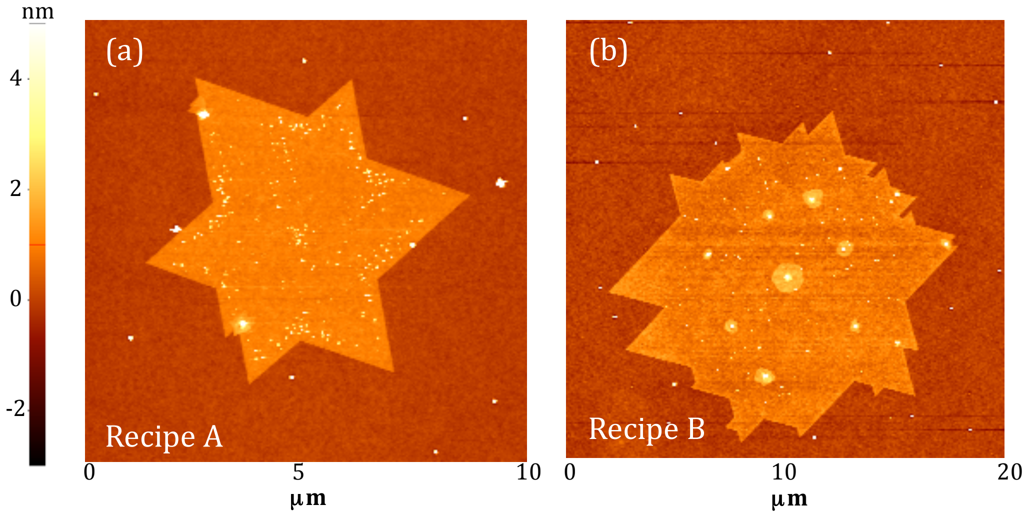

Figure S3: AFM images of WS_2 synthesized using (a) recipe A and (b) recipe B. Monolayer composes 95% and 96% of the growth area in figures (a) and (b) respectively.

As detailed in the main text, the addition of hydrogen in recipe C (T=825 °C) results in substantially different growth characteristics compared to the small isolated islands produced with recipe A and B. Instead, large-area and continuous WS_2 films are synthesized, with isolated growth regions present at the edges of the film. High magnification optical images obtained from multiple samples as well as various regions of the same sample display typical growth characteristics. Each panel in figure S4 displays an area of 125μm x 95μm. Regions of bare substrate, monolayer, and multilayer growth are clearly discernible based on the optical contrast. In addition to uniform monolayer triangles, as displayed in figure 2b of the main text (as well as some of the triangles in S4a and S4d), we observe isolated triangles where multilayer growth is

visually evident. The thicker growth is most often located at the perimeter of the islands (S4b) or regions where triangles merge (indicated by red arrows in S4a), resulting in thin, darker streaks. Similar features are also present in continuous growth regions, and we speculate that these multilayer streaks indicate a boundary line where two growth islands have merged, although additional studies are necessary to confirm this hypothesis. While sample-to-sample variation is present, all samples display a low fraction of multilayer growth, calculated from the contrast in optical images.

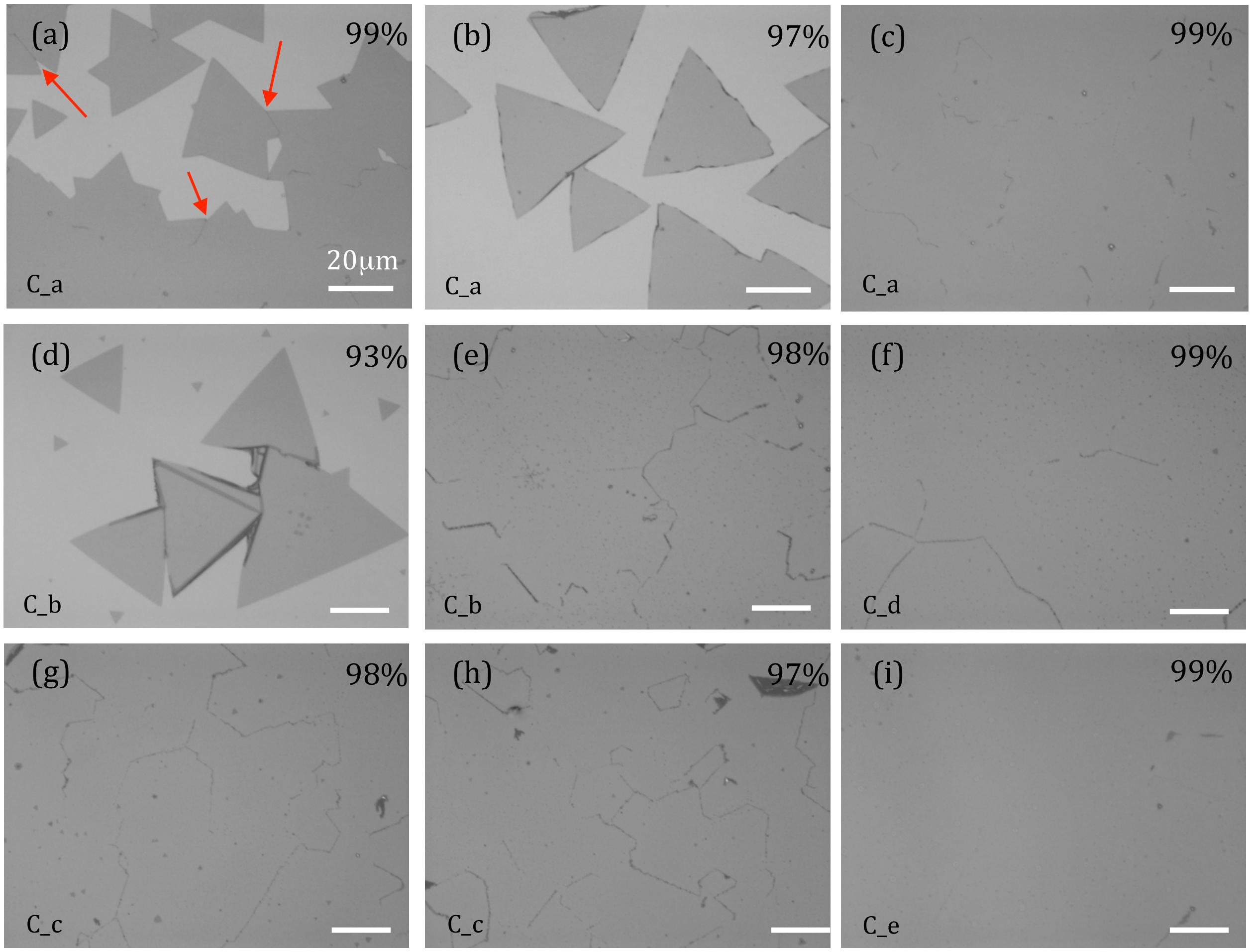

Figure S4: (a-i) Optical images of WS_2 obtained from multiple samples and various regions synthesized using recipe C. The lower case letter identifies the sample under investigation. The percentage of monolayer WS_2 is indicated in the upper right corner. Red arrows in S4(a) indicate darker, multilayer growth that appears to occur where distinct growth regions merge. Scale bar for all images is 20μm.

AFM images of samples synthesized using recipe C provide additional insight into sample quality and structure of multilayer growth. Regions of continuous growth (Figure S5) as well as isolated triangles (Figure S6a) and partially coalesced regions (Figure S6b) are investigated. A small amount of multilayer growth is present in all images. The streak-like features evident in optical images are detected by AFM and typically of bilayer nature. Additional isolated few layer growth regions are evident, as well as small spikes of thick growth regions (~10- 100 nm thick).

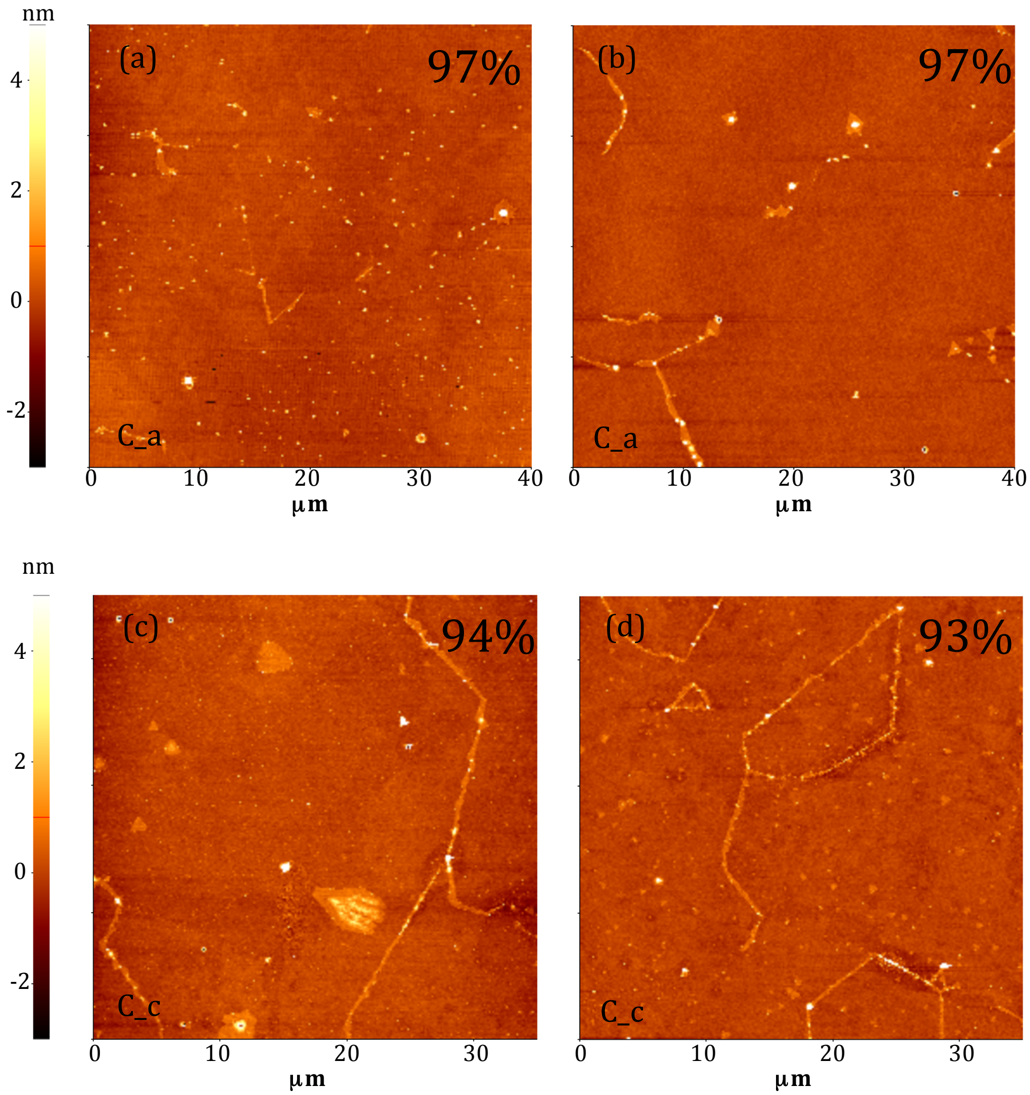

Figure S5 (a-d) AFM images across regions of continuous growth. All are composed mainly of monolayer, with the measured proportion indicated in the upper right corner.

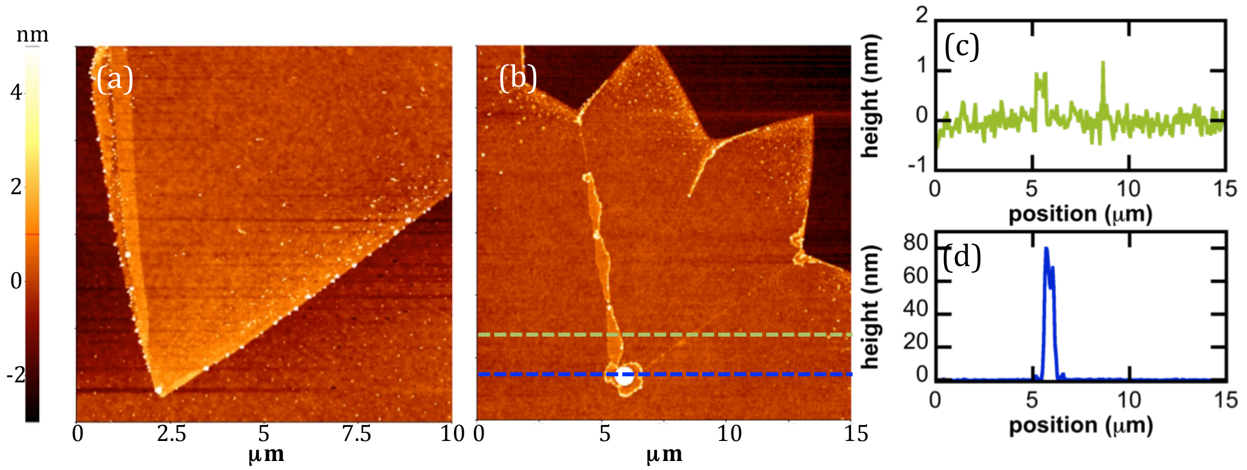

Figure S6 AFM images of the corner of (a) an isolated triangle and (b) a partially coalesced film that was synthesized using Recipe C. The propensity for bilayer growth along the perimeter of triangles and (we presume) where triangles have merged is evident in both (a) and (b). Line scans are taken across (c) a bilayer streak as well as (d) a sharp, thick spike to characterize different modes of multilayer growth.

We performed a post-growth anneal procedure on WS_2 monolayers synthesized using procedure C in order to investigate the effects of pure Ar and Ar/H_2 environments on WS_2. An as-grown WS_2 sample (Figure S7a) is returned to the furnace and exposed to the precise conditions of Recipe A (825°C, Ar=100 sccm), with the exception that no WO_3 precursor is present. As is evident from the optical image of the same area before- and after-anneal, the majority of the WS_2 on the substrate is no longer present following this procedure (Figure S7a). While the thermal stability of a WS_2 monolayer has yet to be determined, bulk WS_2 is stable up to 1250 °C, indicating that thermal decomposition is unlikely. Alternatively, ultrathin layers of TMD materials can succumb to oxidative etching at temperatures below 400 °C.[2,3] While a careful pump-purge cycle is performed prior to annealing, oxygen contamination may potentially enter during the flow of Ar and subsequent oxidative etching could cause the removal of WS_2. This post-growth anneal demonstrates that a pure Ar environment under atmospheric condition is not conducive to high quality WS_2 monolayers. While WS_2 is being formed through the reaction

between S and WO_3 precursors, it is simultaneously being oxidized/ etched. As we observed for highly elevated temperatures (975 °C), the oxidative etching can dominate, resulting in no WS_2 by the conclusion of the synthesis process.

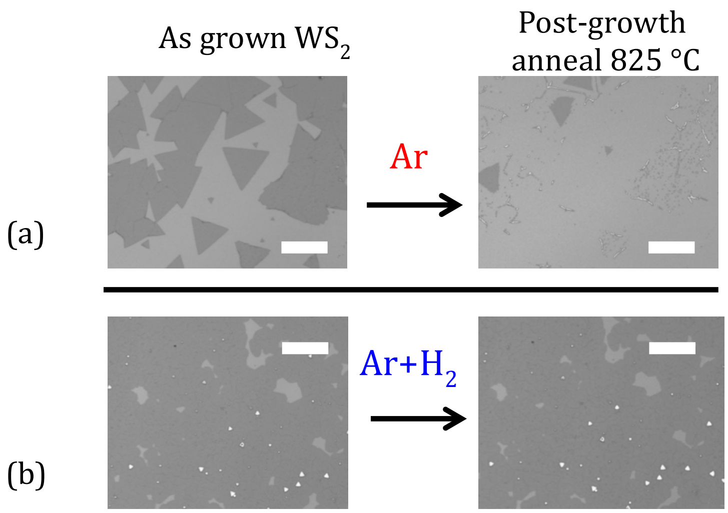

Figure S7: Optical images of the same sample area before-anneal and after-anneal. (a) Exposing as-grown samples to a post-growth anneal at 825°C in pure argon results in the removal of WS_2 monolayers. (b) The addition of hydrogen to the anneal procedure prevents damage to monolayer materials and inhibits oxidative etching of the materials. The scale bar in all images is 100 μm.

In contrast, when the post growth anneal procedure is repeated on a second as-grown sample in a flow of 10 sccm of H_2 and 100 sccm Ar, no oxidative etching occurs, as evident by comparing the before- and after-anneal images in Figure S7b. The WS_2 exhibits no change following the anneal procedure, indicating that hydrogen is inhibiting the presence of destructive oxygen. During the growth of WS_2, the introduction of hydrogen serves multiple purposes: it aids in the formation of WS_2 by reducing the precursor, leading to larger area growth of monolayer material, and it protects against oxygen damage.

References:
1. Ling, X. *et al.* Role of the Seeding Promoter in MoS2 Growth by Chemical Vapor Deposition. *Nano Lett.* **14,** 464–472 (2014).
2. Ionescu, R. *et al.* Oxygen etching of thick MoS2 films. *Chem. Commun.* **50,** 11226–11229 (2014).
3. Zhou, H. *et al.* Thickness-dependent patterning of MoS2 sheets with well-oriented triangular pits by heating in air. *Nano Res.* **6,** 703–711 (2013).
